# The changing face of missed appointments

**DOI:** 10.3399/bjgp23X732249

**Published:** 2023-02-24

**Authors:** Jo Parsons, Gary Abel, Luke TA Mounce, Helen Atherton

**Affiliations:** Unit of Academic Primary Care, Warwick Medical School, University of Warwick, Coventry.; Department of Health and Community Sciences, University of Exeter, Exeter.; Department of Health and Community Sciences, University of Exeter, Exeter.; Associate Professor of Primary Care Research, Warwick Medical School, University of Warwick, Coventry.

## BACKGROUND

Patients failing to attend general practice appointments has substantial time and cost implications for health care. In 2019, approximately 7.2 million GP appointments were missed annually in England, costing the NHS around £216 million.^1^ Missed primary care appointments add to the already over-stretched capacity of GPs and healthcare professionals. In addition to this, patients failing to attend appointments potentially leaves already vulnerable patients living with unmet need, and delays appropriate treatment and diagnoses, adding to longer or more severe conditions with increased cost to the NHS.^2^

A review of studies from five countries found a mean of 15.2% of booked primary care appointments were missed in recent years. Patients of non-white or minority ethnicity, low sociodemographic status, younger age, or with mental health or multiple physical health conditions were more likely to miss appointments.^3^ Common reasons for missing general practice appointments included work or family commitments, forgetting the appointment, difficulties with transportation to get to the appointment, and appointments not being with a preferred GP.^3^

## CHANGES TO GENERAL PRACTICE APPOINTMENTS

The rise of ‘total triage’ approaches in general practice has been well documented in the years leading up to the COVID-19 pandemic^4^ with ‘telephone first’ and ‘digital first’ approaches emerging as a way to manage demand. The COVID-19 pandemic accelerated the implementation of these approaches as they offered a way to help patients remotely where possible, during the national lockdowns. Total triage can be online or over the telephone and usually involves patients waiting for a call back from the practice via telephone regardless of mode of initial contact. Patients are not usually given a fixed appointment time. It is recognised in the research literature that for GPs and practice staff a perceived advantage of a total triage approach is the potential to reduce missed appointments. However, the requirement to make multiple telephone contacts with the patient to achieve this is viewed as a disadvantage.^5^

While the removal of COVID-19 restrictions has seen practices able to reduce the number of remote contacts, there has not been a significant move away from the use of total triage models as appointment systems. There are still more consultations conducted remotely than before the pandemic. In 2022, 37.7% of all general practice appointments were via telephone (a slight increase from 2021 figures), compared with 10% of consultations being via telephone in early 2020.^6^

Overall, the proportion of missed appointments has reduced since the pandemic began and the size of the reduction is approximately comparable between face-to-face and remote modes of consultation.^7^ What is still unknown is whether this reduction can be attributed to changes in access systems, and whether missing a remote contact has different implications for missing a face-to-face contact.

## CHANGES TO MISSED APPOINTMENTS

As documented in the research literature,^3^ information about missed appointments has concerned those booked in advance and missed as a result of an individual either forgetting, or not being able to attend. Interventions to increase appointment adherence, such as providing reminders, have been implemented.^8^ Where consultations are conducted via call-backs, or booked for the same day, reminders ahead of time cannot be sent, and missing the appointment may not be motivated by the same circumstances. Patients may have to wait over a period of a few hours for a telephone call from the healthcare professional, making themselves available over a longer period of time, with the ensuing practicalities involved.

Missed contacts with healthcare professionals via telephone may be the result of a number of factors including:

- poor mobile phone signal;
- telephone lines being used by other people; and
- not being able to have their telephone near them at the time of appointment because of work or other commitments.

Relative to a patient not physically attending a healthcare setting, it is particularly difficult to determine if an unanswered telephone call is a reflection of a patient intentionally not engaging with the appointment, or whether not answering is out of their control. For future service planning, it is important to understand these factors and consider what constitutes non-attendance.

## INCREASED INEQUALITIES?

For some groups of patients, such as those who struggle to attend in-person appointments because of work or travel time,^9^ the shift towards total triage models has increased the accessibility of appointments.^9^ For others, it can increase health inequalities. For example, the need to have a working telephone or internet connection can make it harder to fulfil remote appointments for those who do not have access to them. Telephone consultations are most used by patients with higher levels of education. Patients with higher education qualifications, those with at least one long-term condition, and being female were associated with more awareness and use of online services.^10^

It is not only possible that the group of people missing appointments already may continue to struggle accessing care, but also that additional groups of people are becoming more likely to miss appointments. There is evidence that older people with hearing difficulties, and with caring responsibilities that require them to be present at the consultation, are disadvantaged by a reliance on remote contacts.^11^

Patients from low socioeconomic groups who were previously shown to miss appointments more often^3^ are also less likely to have access to the internet and appropriate technology. Charging patients for missing general practice appointments is often discussed as a possible policy, as an attempt to reduce the rate appointments are missed, but this ignores the evidence available (particularly on those already disadvantaged groups) and ignores the complexities at play in missing appointments.

Currently, there is no research evidence that explores which patients miss appointments now that general practices are widely using total triage with a high proportion of remote contacts compared with when appointments are delivered primarily face-to-face. Other factors have not been explored, for instance, whether the presenting problem impacts on the proportion of appointments that are missed, for example, a difference between long-term condition management and appointments for acute problems. There are many unanswered questions about what a missed appointment looks like and what this means for patient care.

Anecdotally, in recent years general practices have developed their own strategies for managing missed contacts, for instance, agreeing a limited number of call-backs for missed telephone calls or using SMS messaging to contact patients. In the absence of evidence, clear policies about reaching patients could be introduced, and general practices that make changes to their access and consultation systems may wish to monitor the profile of patients who miss appointments to look for change over time and ensure that they are equipped to understand and address any changes to who misses appointments, and why.

Future research should consider all aspects of a missed appointment, at the system level and at the patient level. As long as appointments are missed, there is an opportunity to avoid inequalities and improve delivery of health care; both key aspects of the delivery of general practice.

## References

[1] NHS England (2019). Missed GP appointments costing NHS millions. https://www.england.nhs.uk/2019/01/missed-gp-appointments-costing-nhs-millions/2019/.

[2] Fisher RFR, Croxson CHD, Ashdown HF, Hobbs FDR (2017). GP views on strategies to cope with increasing workload: a qualitative interview study. Br J Gen Pract.

[3] Parsons J, Bryce C, Atherton H (2021). Which patients miss appointments with general practice and why? A systematic review. Br J Gen Pract.

[4] Newbould J, Abel G, Ball S (2017). Evaluation of telephone first approach to demand management in English general practice: observational study. BMJ.

[5] Newbould J, Ball S, Abel G (2019). A ‘telephone first’ approach to demand management in English general practice: a multimethod evaluation. Health Services and Delivery Research.

[6] NHS England GP Patient Survey 2022.

[7] Digital NHS Appointments in general practice November 2019.

[8] George A, Rubin G (2003). Non-attendance in general practice: a systematic review and its implications for access to primary health care. Fam Pract.

[9] Baird B, Maguire D (2021). Understanding factors that enabled digital service change in general practice during the Covid-19 pandemic.

[10] Bryce C, O’Connell MDL, Dale J (2021). Online and telephone access to general practice: a cross-sectional patient survey. BJGP Open.

[11] Bryce C, Fleming J, Parsons J OPTEL — older people and ‘telephone access’ to general practice.

